# Long noncoding RNA DANCR expression and its predictive value in patients with atherosclerosis

**DOI:** 10.1080/21655979.2022.2033408

**Published:** 2022-03-02

**Authors:** Fengxia An, Yanliang Yin, Weixian Ju

**Affiliations:** Department of Health, Dongying People’s Hospital, Shandong, China

**Keywords:** Atherosclerosis, lncRNA DANCR, vascular smooth muscle cells, diagnosis, miR-335-5p

## Abstract

Long noncoding RNAs (lncRNAs) act crucial roles in the progression of vascular diseases, including atherosclerosis. This study aims to investigate the expression levels of the atherosclerosis-associated lncRNA DANCR in patients diagnosed with atherosclerosis and whether its abnormal expression affects the progress of atherosclerosis. The expression of DANCR in the serum samples of all study participants was quantified using RT-qPCR. Then, the predictive capacities of DANCR for the detection of atherosclerosis patients were evaluated via receiver operating characteristic (ROC) curve analysis. The effects of DANCR on vascular smooth muscle cells (VSMCs) proliferation and migration were then explored using cell counting kit-8 (CCK-8) and Transwell migration assays. The DANCR exhibited increased expression trends in patients with atherosclerosis than healthy controls. Moreover, there were differences in the levels of low-density lipoprotein cholesterol (LDL-C), homocysteine (Hcy), and C-reactive protein (CRP) between the healthy controls and atherosclerosis patients. The DANCR expression was positively correlated with serum LDL-C, Hcy, and CRP levels. DANCR expression could distinguish patients with atherosclerosis from healthy individuals with a high area under the ROC curve (AUC), sensitivity, and specificity. Additionally, knockdown of DANCR weakened the proliferative abilities and migration capacities of VSMCs. It was also shown that DANCR could compete with miR-335-5p binding. Herein, it appears that the LncRNA DANCR was closely associated with the progression of atherosclerosis by targeting miR-335-5p, which might be a potential detective predictor and target for the treatment of atherosclerosis.

## Graphical abstract

**Figure:**
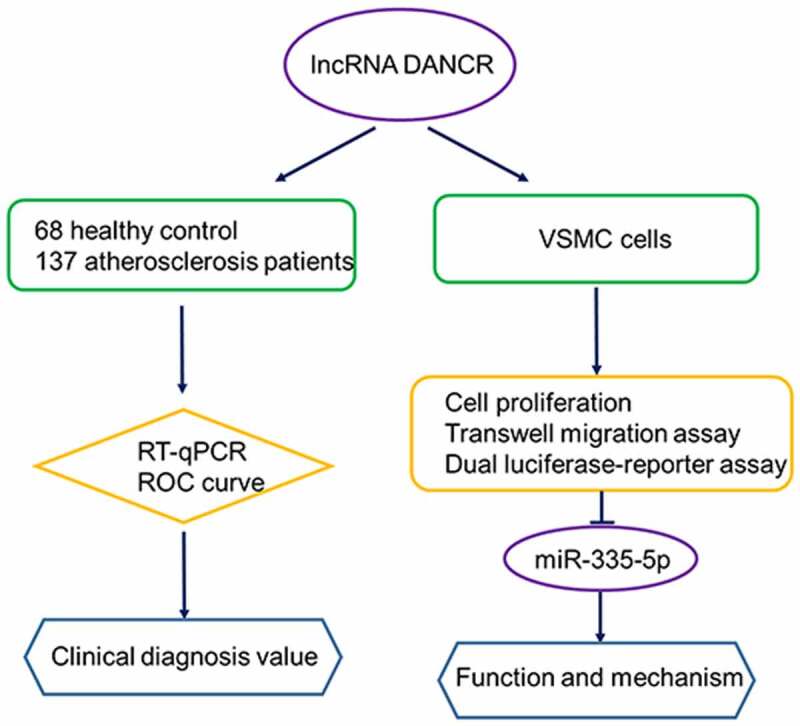

## Introduction

Atherosclerosis underlies the vast majority of common cardiovascular disorders, including coronary artery disease and stroke, which is one of the leading causes of mortality burden worldwide [1]. Carotid atherosclerosis is a manifestation of systemic atherosclerosis in the carotid arteries mainly through the formation of atherosclerotic plaques. These plaques can lead to carotid artery lumen stenosis and hardening, resulting in the increased risk of cerebral infarction [2]. The viability, migration, and accumulation of vascular smooth muscle cells (VSMCs) are essential factors in the formation of stable plaques during the progression of atherosclerosis [3]. Therefore, early diagnosis of atherosclerosis and its underlying its molecular mechanism is essential for the therapeutic strategy for atherosclerosis.

Non-coding RNAs include various types such as microRNA (miRNA), small nuclear RNA, and long noncoding RNA (lncRNAs) [4]. Among these noncoding RNAs, lncRNAs are approximately 200 nt long, and their specific changes of expression are involved in the pathological process of multiple diseases [5–7]. The abnormal expression of lncRNAs can serve as biomarkers throughout the progression of the disease [8–10]. In atherosclerosis, several lncRNAs are involved in its progression and act as diagnostic or prognostic biomarkers, such as lncRNA MALAT1 [11], lncRNA XIST [12], and lncRNA TCONS_00034812 [13]. A recent study indicated that the lncRNA DANCR had aberrant expression levels in patients with coronary artery disease [14]. However, the clinical and functional role of DANCR with involvement in atherosclerosis disease pathogenesis remains unclear.

This study aims to investigate the clinical role and functional role of DANCR in the pathogenesis of atherosclerosis and explore the potential molecular mechanisms, wherein the expression pattern of DANCR was measured in the serum of patients with atherosclerosis and healthy controls. Then, the loss-of-function experiments were conducted to test the proliferative abilities and migration capacities of VSMC cells. After that, the potential sponge of DANCR was uncovered in atherosclerosis.

## Materials and methods

### Study population

All experimental procedures were approved by the Ethics Committee of Dongying People’s Hospital (approval No.DYYX-2018-026). The study population consisted of 68 healthy individuals and 137 patients with atherosclerosis, and all the patients and healthy individuals signed written informed consent.

From January 2018 to September 2020, the participants were consecutively recruited from Dongying People’s Hospital. The inclusion criteria include: 1) atherosclerosis patients diagnosed with plaque formation using carotid artery ultrasonography. 2) Patients have complete clinical characteristics. The exclusion criteria include: 1) patients with previous myocardial infarction and percutaneous coronary intervention. 2) uncontrolled malignant arrhythmia. 3) patients with acute and chronic infectious diseases and malignant tumors. 3) patients with severe liver and kidney diseases. The healthy subjects who received annual routine physical examination in our hospital were enrolled in healthy control group. The healthy control and patient groups were matched for age, sex, and body mass index (BMI). The clinical characteristics and analysis results were then collected and recorded.

### Serum collection and storage

Blood samples were obtained from the study population and were centrifuged at 1000 × g for 15 min to collect the serum. The serum samples were then transferred to RNase/Dnase-free 2-mL EP tubes and stored at −80°C until RNA extraction.

### RNA extraction and real-time quantitative PCR

Total RNA from serum samples and cells were isolated using RNAiso Plus (TaKaRa, Otsu, Shiga, Japan) and then reverse-transcribed into cDNA using a PrimeScript real-time (RT) reagent kit (Takara Bio, Japan). The real-time quantitative PCR (RT-qPCR) was carried out using either a SYBR Green qPCR SuperMix (Invitrogen, USA) or a TaqMan MicroRNA assay kits (Applied Biosystems, USA) on an ABI PRISM7500 Sequence Detection System [15]. The expression pattern of DANCR and miR-335-5p were then calculated using the 2^−ΔΔCt^ (cycle threshold) method and were normalized to the expression of GAPDH and U6, respectively.

The sequences for PCR were as follows: DANCR, 5’- ACCCAGGCTGGATGGAGTAT-3’ (forward) and 5’-CAGCTGGCTACAACAGAGCT-3’ (reverse); miR-335-5p, 5’-GGGTCAAGAGCAATAACGA-3’ (forward) and 5’-CTCAACTGGTGTCGTGGA-3’ (reverse); GAPDH, 5’-TCATCTCTGCCCCCTCTGCT-3’ (forward) and 5’-CGACGCCTGCTTCACCACCT-3’ (reverse); and U6, 5’-GCTTCGGCAGCACATATACTAAAAT-3’ (forward) and 5’-CGCTTCACGAATTTGCGTGTCAT-3’ (reverse).

### Cell culture and transfection

A human vascular smooth muscle cell line (VSMC; CRL-1999) was purchased from the American Type Culture Collection (ATCC) and cultured as per the manufacture’s instructions. The cells were cultured in a DMEM medium (Gibco, CA, USA) added with 10% fetal bovine serum (FBS; Gibco) and were incubated at 37°C in an incubator with 5% CO_2_.

To adjust the DANCR expression and explore its effects on VSMC cells, DANCR siRNA (si-DANCR; 5’-GCUGGUAUUUCAAUUGACUTT-3’) or siRNA negative control (si-NC, 5’-UUCUCCGAACGUGUCACGUTT-3’; RiboBio, Guangzhou, China) was transfected into VSMC cells using Lipofectamine 2000 (Invitrogen, Carlsbad, CA, USA). At indicated time point (24 h) post the transfection, cells were harvested for further cellular experiments analysis.

### Cell counting kit-8 assay

The cell counting kit-8 (CCK-8; Donjindo, Japan) was used to determine cell viability [16]. Briefly, VSMC cells (2000 cells/well) were plated in 96-well plates and cultured at 37°C. Then, 10 μl CCK-8 solution was added to each well at different time points (0, 24, 48, 72 h), and the cells were incubated for another 2 h. Subsequently, the optical density of the samples was detected at 450 nm using a microplate absorbance reader (Bio-Tek, Elx800, USA).

### Transwell migration assay

The migratory abilities of VSMC cells treated with si-DANCR were measured using a 24-well Transwell migration assay [17]. Briefly, transfected VSMC cells (3 × 10^4^ cells) were re-suspended in a serum-free medium and seeded into the top chamber, and complete medium with 10%FBS was added to the the lower chambers. After 24 h, the migrated cells on the membrane were fixed with 4% paraformaldehyde and stained with 0.5% crystal violet. The numbers of cells per field was then counted under a light microscope in five random fields.

### Dual-luciferase reporter assay

The LncBase Experimental v.2 online database (http://carolina.imis.athena-innovation.gr/diana_tools/web/index.php?r=lncbasev2%2findex-experimental) was used to predict the binding sites between DANCR and miR-335-5p. Dual-luciferase reporter assay was used to validate the correlation between DANCR and miR-335-5p [18]. Briefly, the wild-type (wt) or mutant (mut) 3’-UTR fragments of DANCR were then amplified and cloned into the firefly luciferase in the PGL3 vector (Promega, Madison, WI, USA). The VSMC cells were then cultured 24 h after being seeded into a 24-well plate and co-transfected with the WT-DANCR or MUT-DANCR and miR-335-5p mimic or miR-335-5p inhibitor. After 48 h post-transfection, firefly luciferase activities were detected using a Dual-Luciferase Reporter Assay System (Promega) and were normalized to the Renilla luciferase activities.

### Statistical analysis

All the results are expressed as the mean ± SD from at least three independent experiments or replicate. Data were checked for normality via the Kolmogorov–Smirnov (K-S) normality test. All statistical analyses were carried out using SPSS 20.0 (SPSS software, Chicago, IL, USA) and GraphPad Prism 7.0 software (GraphPad Software, La Jolla, CA, USA). Differences between groups were compared through the unpaired student’s t-test or one-way ANOVA. Correlations of the datasets were assessed using Pearson’s correlation coefficient r analysis. The difference was considered as statistically significant when the *P*-value was lower than 0.05.

## Results

In the current study, the expression of DANCR was detected in the serum samples of all participants using RT-qPCR. The diagnostic power of DANCR in atherosclerosis patients was analyzed using the receiver operator characteristic curve (ROC) curve. The effects of DANCR on cell viability and migration in VSMC cells were explored after transfection of si-DANCR or si-NC using CCK-8 assay and Transwell migration assay. The potential target miRNAs of lncRNAs were evaluated and confirmed using LncBase Experimental v.2 online bioinformatics database and dual-luciferase reporter assay.

### The demographic and clinical characteristics of participants

The demographic characteristics and clinical characteristics of the subjects are provided in Table 1. The age of the atherosclerosis patients was 49.07 ± 4.38 years and the age of healthy controls was 48.95 ± 4.49 years. The atherosclerosis patients include 75 males and 62 females, and the healthy controls include 37 males and 31 females. The BMI was 22.87 ± 3.37 kg/m^2^ and 22.16 ± 2.83 kg/m^2^ of atherosclerosis patients and the healthy control group, respectively. Thus, the demographic information has no statistical difference between atherosclerosis patients and healthy control. The levels of low-density lipoprotein cholesterol (LDL-C), homocysteine (Hcy), and C-reactive protein (CRP) exhibited significant differences between the healthy control group and patients with atherosclerosis (*P* < 0.05). The serum total cholesterol (TC), high-density lipoprotein cholesterol (HDL-C), fasting blood glucose (FBG), triglyceride (TG), aspartate aminotransferase (AST), alanine aminotransferase (ALT), uric acid (UA), and creatinine (Cre) levels were not significantly different between groups (*P* > 0.05).

**Table 1.** The difference in clinical data of the study population

Features	Healthy controls (n = 68)	AS patients (n = 137)	*P* value
Age (years)	48.95 ± 4.49	49.07 ± 4.38	0.850
Sex (male/female)	37/31	75/62	0.964
BMI (kg/m^2^)	22.16 ± 2.83	22.87 ± 3.37	0.117
TC (mmol/L)	4.03 ± 0.80	4.24 ± 1.09	0.153
HDL-C (mmol/L)	1.59 ± 0.56	1.63 ± 0.50	0.647
LDL-C (mmol/L)	2.86 ± 0.85	3.12 ± 0.83	0.042
TG (mmol/L)	1.36 ± 0.086	1.38 ± 0.077	0.089
FBG (mmol/L)	4.95 ± 0.50	4.93 ± 0.59	0.785
ALT (U/L)	18.35 ± 8.72	20.21 ± 10.14	0.175
AST(U/L)	19.79 ± 10.16	21.08 ± 10.55	0.398
UA (mm Hg)	247.85 ± 64.04	251.44 ± 65.62	0.708
Cre (mm Hg)	69.83 ± 21.36	70.94 ± 19.15	0.718
Hcy (μmol/L)	10.67 ± 2.45	11.46 ± 2.35	0.030
CRP (mg/l)	6.13 ± 1.89	19.56 ± 3.22	<0.001

Note: TC: Total cholesterol; HDL-C, high-density lipoprotein cholesterol; LDL-C, low-density lipoprotein cholesterol; TG: triglyceride; FBG, fasting blood glucose; ALT, alanine aminotransferase; AST, aspartate aminotransferase; UA, uric acid; Cre, serum creatinine; Hcy, homocysteine; CRP, C-reactive protein.

### Serum DANCR expression levels and clinical value in patients with atherosclerosis

**Figure 1.**
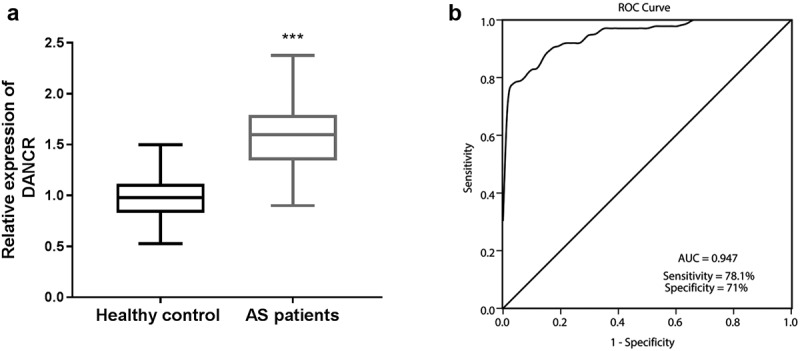
DANCR levels in the serum of patients with atherosclerosis. (a) The serum DANCR was highly expressed in the serum of patients with atherosclerosis compared to healthy controls. (*P* < 0.001). (b) ROC curve analysis showed that DANCR could distinguish patients with atherosclerosis from healthy individuals. The AUC was 0.947, a sensitivity of 78.1%, and a specificity of 71%.

The DANCR expression levels were detected in the serum samples of all participants through RT-qPCR. As displayed in Figure 1(a), DANCR showed a significant trends toward stepwise increases (about 1.6-fold) from the healthy control group (0.977 ± 0.199) to the atherosclerosis patients group (1.586 ± 0.327).

To further assess the diagnostic power of DANCR in patients with atherosclerosis, the ROC curve was conducted to estimate the sensitivity and specificity of DANCR expression for distinguishing patients with atherosclerosis from healthy participants. As exhibited by Figure 1(b), DANCR displayed a strong differentiation power between patients with atherosclerosis and healthy individuals. The area under the ROC curve (AUC) of DANCR was 0.947, with a sensitivity of 78.1% and a specificity of 71%.

**Figure 2.**
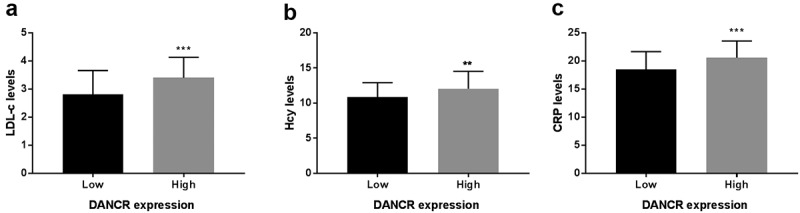
Association between DANCR expression and LDL-C, Hcy, and CRP levels. (a) LDL-C levels were higher in the high DANCR expression group than the low DANCR expression group. (b) Hcy levels were high in the high DANCR expression group. (c) DANCR expression was positively associated with CRP levels. **P < 0.01, ***P < 0.001.

Based on the median serum DANCR expression level, patients with atherosclerosis were grouped into the low- and high- DANCR expression groups. Furthermore, on account of the statistical difference of LDL-C, Hcy, and CRP between the healthy control group and atherosclerosis patient group, the corerlation between DANCR expression and LDL-C, Hcy, or CRP levels was analyzed using Pearson correlation analysis, the LDL-C, Hcy, and CRP levels were evaluated in the low- and high-expression DANCR groups. Firstly, the correlation between were analyzed using Pearson correlation analsis. The results in Table 2 showed that DANCR expression levels were positively correlated with LDL-C (pearson r = 0.2977, 95%CI 0.1369–0.4433, *P* = 0.0004), Hcy (pearson r = 0.2276, 95%CI: 0.0623–0.3808, *P* = 0.0075), or CRP levels (pearson r = 0.3217, 95%CI: 0.1627–0.4643, *P* = 0.0001). As exhibited in Figures 2(a-c), LDL-C, Hcy, and CRP levels were elevated in the high DANCR expression group relative to the low DANCR expression group (*P* < 0.01). These results demonstrated that elevated LDL-C, Hcy, and CRP levels might increase the risk of atherosclerosis and were positively correlated with DANCR expression levels.

**Table 2.** The correlation analysis between DANCR expression and risk factors of atherosclerosis

	DANCR expression		
Parameters	Pearson r	95%CI	*P*-values
LDL-C	0.2977	0.1369–0.4433	0.0004
Hcy	0.2276	0.0623–0.3808	0.0075
CRP	0.3217	0.1627–0.4643	0.0001

### Knockdown of DANCR attenuated the proliferation and migration of VSMC cells

**Figure 3.**
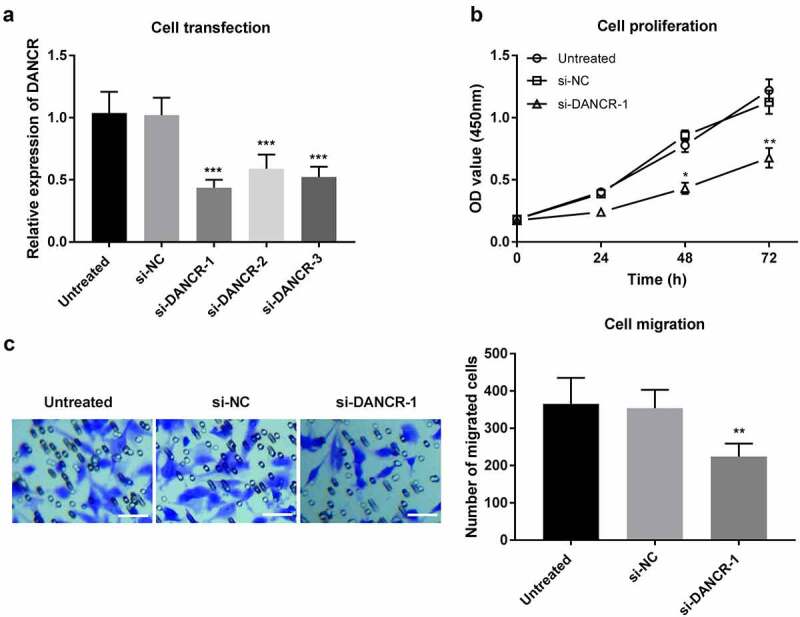
Regulation of DANCR on proliferation and migration of VSMC cells. (a) Expression of DANCR in VSMC cells transfected with siRNA or the negative control. (b) The proliferation of VSMC cells was measured using a CCK-8 assay after DANCR knockdown. (c) The migration of VSMC cells was detected through a Transwell migration assay after DANCR knockdown. (Scale bar, 50 μm) *P < 0.05, **P < 0.01, ***P < 0.001.

To evaluate the functional potential of DANCR in patients with atherosclerosis, VSMCs, which play a crucial role in atherosclerosis, were used in the in vitro experiments. The effects of DANCR on cell viability and migration in VSMC cells were explored after transfection with si-DANCR or si-NC. As displayed in Figure 3(a), the expression of DANCR was significantly downregulated by si-DANCR (*P* < 0.001). The CCK-8 assay then revealed that DANCR knockdown could attenuate the proliferation ability of VSMCs (*P* < 0.05, Figure 3(b)). Additionally, Transwell migration assay showed that silence of DANCR receded the migration ability in VSMCs (*P* < 0.01, Figure 3(c)). These data revealed that DANCR knockdown could attenuate the proliferation and migration of VSMC cells, suggesting that DANCR might play a role in accelerating the formation of atherosclerosis.

### miR-335-5p, a target of DANCR

**Figure 4.**
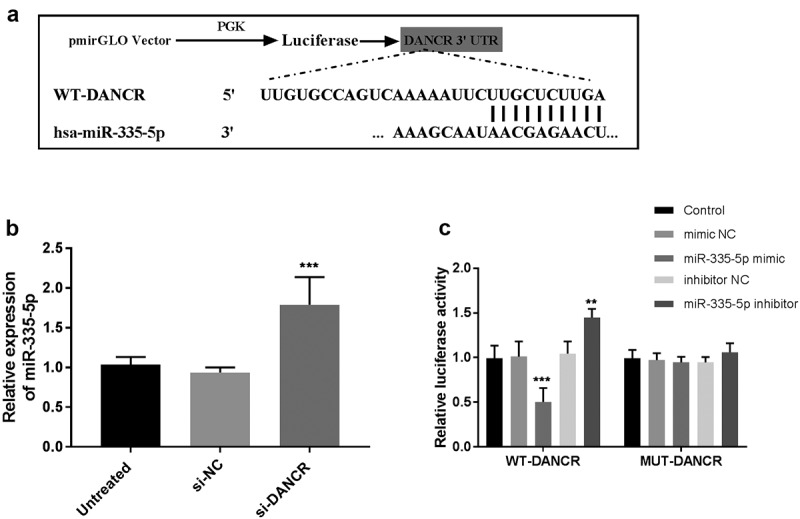
miR-335-5p is a target of DANCR in VSMC cells. (a) Bioinformatics analyses predicted the binding sites between DANCR and miR-335-5p. (b) The RT-qPCR was conducted to detect miR-335-5p expression in VSMC cells transfected with DANCR siRNA. (c) Dual-luciferase reporter assay validated the interaction between DANCR and miR-335-5p. **P < 0.01, ***P < 0.001.

Many studies have demonstrated that cytoplasmic lncRNAs can serve as miRNA sponges. The LncBase Experimental v.2 online bioinformatics database was used to identify the potential miRNA recognition elements and the binding sites on DANCR. Among the potential miRNAs, miR-335-5p could reduce atherosclerotic vulnerable plaque formation in acute coronary syndrome [19] and be differentially expressed in stroke [20], thus, miR-335-5p was selected to confirm the interferes with DANCR. The sequences of 3’-UTR of DANCR along with the miR-335-5p binding sites are shown in Figure 4(a). Subsequently, the expression of miR-335-5p was detected in VSMC cells that were transfected with si-DANCR. The results then indicated that miR-335-5p expression significantly increased in the DANCR-knockdown VSMC cells (*P* < 0.001, Figure 4(b)). The dual-luciferase reporter assay revealed that co-transfection with DANCR-WT and miR-335-5p mimic suppressed luciferase activity, while the addition of miR-335-5p inhibitor increased luciferase activity (*P* < 0.01, Figure 4(c)). However, co-transfection with DANCR-mut and miR-335-5p mimic or inhibitor failed to affect luciferase activity (Figure 4(c)). Therefore, the data revealed that a direct interaction between DANCR and miR-335-5p.

## Discussion

Atherosclerosis is a persistent inflammatory condition, combined with its accompanying complications (such as stroke, myocardial infarction, and artery diseases), contributing to the occurrence of cardiovascular diseases [21]. In the current study, DANCR was found to be highly expressed in the serum of patients with atherosclerosis. The LDL-C, Hcy, and CRP levels exhibited statistical differences between the healthy controls and patients with atherosclerosis and were also associated with serum DANCR expression levels. Further, DANCR knockdown could decrease the proliferation and migration abilities of VSMCs by sponging miR-335-5p.

Some studies have demonstrated that non-coding RNAs have a crucial role in the process of atherosclerosis [12,22]. For instance, knockdown of the lncRNA NORAD promoted oxidized low-density lipoprotein-induced endothelial cell senescence and atherosclerosis [23].Similarly, the LncRNA SNHG14 could promote atherosclerosis by regulating the VSMC cell apoptosis/proliferation balance in atherosclerosis by sponging miR-19a-3p [24]. In other studies, DANCR was reported to have an oncogenic role and was associated with the progression of various tumors [25], including lung cancer [26] and ovarian cancer [27]. It was also observed that DANCR was aberrantly expressed in peripheral blood mononuclear cells of patients with coronary artery disease and obstructive coronary atherosclerosis subjects [14]. However, the detailed role of DANCR in atherosclerosis remains elusive. In the present study, DANCR was shown to be highly expressed in the serum of patients with atherosclerosis compared to healthy controls, suggesting that DANCR may promote the process of atherosclerosis. A similar role of DANCR was observed in cardiomyocytes, as its upregulation of DANCR could inhibit apoptosis and promote autophagy to protect cardiomyocytes from endoplasmic reticulum stress that leads to the conduces to the etiopathogenesis of myocardial infarction [28]. Additionally, it was observed that DANCR was significantly associated with LDL-C, Hcy, and CRP levels, which are well-known risk factors for atherosclerosis and related cardiovascular diseases [29,30]. These data then implied that DANCR may be a risk factor and involved in the process of atherosclerosis.

Previous studies highlights the potential of lncRNAs could serve as clinical diagnostic or prognostic markers in multiple diseases [31,32]. Considering the differential expressions of DANCR between atherosclerosis patients and healthy individuals, we further analyzed whether DANCR has clinical predictive value using an ROC curve. The high AUC, sensitivity, and specificity revealed that DANCR could be a diagnostic marker for distinguishing atherosclerosis patients from healthy individuals. Previous studies also reported that DANCR both diagnosis and prognosis prediction value in many types of cancers [33], such as colorectal cancer [34]. These studies and results revealed that DANCR may be a potential diagnostic marker for patients with atherosclerosis.

The proliferation and migration of VSMC cells have essential roles in the progression of atherosclerosis. For instance, the viability and migration of VSMC cells could be inhibited by LOC285194, which might be a therapeutic target to treat atherosclerosis [3]. Downregulation of lncRNA SNHG8 suppressed VSMC cell proliferation and migration, which also had diagnostic value for atherosclerosis [35]. Loss-of-function assays indicated that DANCR knockdown weakened the proliferation and migration of VSMC cells, implying that downregulation of DANCR may contribute to the prevention of atherosclerosis. Interestingly, a recent study indicated that DANCR was downregulated in calcifying VSMC cells compared to non-calcified VSMC cells, showing that DANCR might mediate VSMC calcification [36]. A previous study demonstrated that overexpression of DANCR promoted cell viability, migration, and angiogenesis in oxygen-glucose deprivation-treated brain microvascular endothelial cells through the miR-33a-5p/XBP1s [37]. DANCR could play crucial roles in various diseases through sponging miRNAs, such as miR-214-5p [38], miR-19a-3p [39], miR-135b-5p [40], and miR-335-5p [41,42]. Among these potential target miRNAs, it is observed that miR-335-5p was a sponge of DANCR in the current study. A recent study revealed that upregulation of miR-335-5p could reduce atherosclerotic vulnerable plaque formation in acute coronary syndrome by targeting Jagged1 (JAG1) through regulating Notch signaling [19]. These results suggest that DANCR may regulate atherosclerosis by targeting miR-335-5p. Based on the previous study and present data, it is speculated that DANCR may involved in the progression of atherosclerosis through miR-335-5p/JAG1/Notch signaling.

However, there are some limitations in the current study. Firstly, the disparity in sample size between atherosclerosis patients and healthy control was a limitation that strains the statistical analysis in the preliminary study. On the other hand, the effects of DANCR on cellular activities were explored using VSMC cells. Future studies will use more cells that contribute to atherosclerotic lesion formation (such as endothelial cells) to evaluate the role of DANCR in atherosclerosis. Finally, the detailed mechanism of DANCR in atherosclerosis needs to be further explored in future studies.

## Conclusion

LncRNA DANCR was highly expressed in the serum samples of patients with atherosclerosis and was positively associated with LDL-C, Hcy, and CRP levels. DANCR could be a potential diagnostic marker to distinguish atherosclerosis patients from healthy individuals. Knockdown of DANCR could reduce proliferation capacities and migration abilities of VSMC cells through the regulation of miR-335-5p. This study uncovered that DANCR might be a diagnosis predictor and a potential target for treating atherosclerosis.
